# Mitochondrial Dysfunction and Its Potential Molecular Interplay in Hypermobile Ehlers–Danlos Syndrome: A Scoping Review Bridging Cellular Energetics and Genetic Pathways

**DOI:** 10.3390/cimb47020134

**Published:** 2025-02-19

**Authors:** Purusha Shirvani, Arash Shirvani, Michael F. Holick

**Affiliations:** Ehlers-Danlos Syndrome Clinical Research Program, Section of Endocrinology, Diabetes, Nutrition and Weight Management, Department of Medicine, Boston University Chobanian & Avedisian School of Medicine, Boston, MA 02118, USA; shirvani@bu.edu

**Keywords:** hypermobile Ehlers–Danlos Syndrome, mitochondrial dysfunction, oxidative phosphorylation, reactive oxygen species, genetic mutations, molecular pathways, extracellular matrix, mitochondrial DNA

## Abstract

Hypermobile Ehlers–Danlos Syndrome (hEDS) is a hereditary connective tissue disorder characterized by joint hypermobility, skin hyperextensibility, and systemic manifestations such as chronic fatigue, gastrointestinal dysfunction, and neurological symptoms. Unlike other EDS subtypes with known genetic mutations, hEDS lacks definitive markers, suggesting a multifactorial etiology involving both mitochondrial dysfunction and non-mitochondrial pathways. This scoping review, conducted in accordance with the PRISMA-ScR guidelines, highlights mitochondrial dysfunction as a potential unifying mechanism in hEDS pathophysiology. Impaired oxidative phosphorylation (OXPHOS), elevated reactive oxygen species (ROS) levels, and calcium dysregulation disrupt cellular energetics and extracellular matrix (ECM) homeostasis, contributing to the hallmark features of hEDS. We reviewed candidate genes associated with ECM remodeling, signaling pathways, and immune regulation. Protein–protein interaction (PPI) network analyses revealed interconnected pathways linking mitochondrial dysfunction with these candidate genes. Comparative insights from Fabry disease and fragile X premutation carriers underscore shared mechanisms such as RNA toxicity, matrix metalloproteinases (MMP) activation, and ECM degradation. These findings may suggest that mitochondrial dysfunction amplifies systemic manifestations through its interplay with non-mitochondrial molecular pathways. By integrating these perspectives, this review provides a potential framework for understanding hEDS pathogenesis while highlighting latent avenues for future research into its molecular basis. Understanding the potential role of mitochondrial dysfunction in hEDS not only sheds light on its complex molecular etiology but also opens new paths for targeted interventions.

## 1. Introduction

Hypermobile Ehlers–Danlos Syndrome (hEDS) stands out as the most common form of EDS, yet it remains frequently underdiagnosed due to its diverse clinical manifestations and the absence of specific genetic markers [1,2]. This hereditary connective tissue disorder is characterized by joint hypermobility, tissue fragility, and wide-ranging systemic effects [1,2]. Recent epidemiological data suggest hEDS may be more prevalent than previously thought [3,4]. While global estimates indicate that EDS affects approximately 1 in 5000 individuals [3], localized studies in Wales have reported a much higher incidence of 1 in 500 for hEDS and related Hypermobility Spectrum Disorders (HSDs) [4]. Notably, females account for about 70% of diagnosed cases, potentially due to estrogen’s influence on collagen metabolism and pain perception [5,6,7,8]. hEDS exhibits an autosomal dominant inheritance pattern with significant phenotypic variability within families [9,10]. This heterogeneity, coupled with the lack of definitive biomarkers, often leads to prolonged diagnostic delays [11]. Unlike other EDS variants with known collagen gene mutations, hEDS’s genetic basis remains elusive [1,2]. Current research explores potential links to genes involved in extracellular matrix (ECM) regulation, mitochondrial function, and neuromuscular signaling [2]. This genetic complexity underscores the need for a multifaceted approach to understanding hEDS pathophysiology, integrating connective tissue biology with broader systemic processes. Recent advances in molecular biology and cellular energetics have highlighted mitochondrial dysfunction as a potential key player in hEDS pathophysiology [2,5]. Mitochondria play a crucial role in maintaining connective tissue homeostasis through several key mechanisms. Firstly, mitochondrial oxidative phosphorylation (OXPHOS) provides the ATP necessary for collagen biosynthesis, post-translational modifications, proteoglycan synthesis, ECM assembly, and fibroblast migration during tissue repair [2,5,12]. Secondly, mitochondria regulate redox balance, with balanced ROS production maintaining ECM integrity by modulating matrix metalloproteinases (MMPs) and their inhibitors (TIMPs), while excessive mitochondrial reactive oxygen species (ROS) can lead to collagen fragmentation and elastin degradation [13,14,15]. Thirdly, mitochondrial calcium buffering is essential for integrin-mediated cell–ECM interactions, Transforming Growth Factor Beta (TGF-β) signaling in fibroblast activation, and smooth muscle contractility [16,17]. Recent research has shown the potential role of mitochondrial DNA (mtDNA) mutations and mitochondrial dysfunctions in the development of connective tissue disorders [18,19,20]. This discovery may suggest a novel mechanism linking mitochondrial dysfunction to the widespread connective tissue abnormalities observed in disorders such as hEDS, opening new avenues for understanding the complex interplay between mitochondrial function and connective tissue homeostasis. Additionally, sex-specific modulation occurs through estrogen–mitochondria interactions via ERα receptors, influencing collagen cross-linking enzymes and hyaluronic acid synthesis [21], possibly explaining the 3:1 female predominance in hEDS [6,7,8]. MtDNA inheritance patterns and sex-specific hormonal influences on mitochondrial function could contribute to this sexual dimorphism [22]. Mitochondrial dysfunction can potentially impair connective tissue in hEDS through bioenergetic failure, oxidative stress, and calcium mishandling, leading to reduced ECM component synthesis, the degradation of collagen and elastin networks, and disrupted cellular signaling [12,13,14,15,16,17]. Proteomic analyses have revealed significant alterations in mitochondrial proteins involved in OXPHOS, including ATP synthase components and enzymes critical for energy metabolism [23].

Mitochondrial dysfunction may also underlie several poorly understood aspects of hEDS, including chronic fatigue, gastrointestinal dysfunction, and neurological manifestations [5,24]. Recent studies have demonstrated that impaired mitochondrial energy production correlates with exercise intolerance and post-exertional malaise in hEDS patients [25,26]. The high energy demands of the gastrointestinal smooth muscle and enteric nervous system make them particularly vulnerable to mitochondrial dysfunction [27].

This review examines the emerging evidence linking mitochondrial dysfunction to hEDS pathophysiology, analyzing recent advances in understanding how mitochondrial abnormalities can potentially influence connective tissue homeostasis, energy metabolism, and cellular signaling pathways.

## 2. Materials and Methods

This review utilized a systematic and comprehensive approach to identify, evaluate, and synthesize existing evidence on the role of mitochondrial dysfunction in the pathogenesis of hEDS. The methodology was designed to ensure a rigorous selection of high-quality studies and provide a robust foundation for exploring the interplay between mitochondrial pathways and hEDS pathophysiology. This scoping review was conducted in accordance with the PRISMA-ScR guidelines (supplementary material) to ensure comprehensive reporting and methodological rigor [28].

### 2.1. Literature Search Strategy

A systematic search was conducted across four major databases: PubMed, Web of Science, Google Scholar, and Scopus. The search terms included combinations of keywords such as “*Mitochondria*”, “*Mitochondrial dysfunction*”, “*Mitochondrial pathway*”, “*Mitochondrial mutation*”, “*hypermobile Ehlers-Danlos Syndrome*”, “*Ehlers-Danlos Syndrome type 3*”, and “*hEDS*”. Boolean operators (AND/OR) were used to refine the search strategy. The search was not restricted by publication year but was limited to peer-reviewed articles published in English.

#### 2.1.1. Inclusion Criteria

The eligibility criteria for study inclusion were as follows:

• Studies explicitly addressing mitochondrial dysfunction, mitochondrial mutations, or mitochondrial pathways in the context of hEDS.
• Articles exploring genetic mutations linked to mitochondrial or ECM pathways relevant to hEDS.
• Primary research articles, case studies, or experimental studies with original data.

#### 2.1.2. Exclusion Criteria

• Review articles, books, or conference abstracts without original data.
• Studies not published in English.
• Articles focused on unrelated EDS subtypes or conditions without relevance to mitochondrial dysfunction.

#### 2.1.3. Study Selection Process

**Figure 1:**
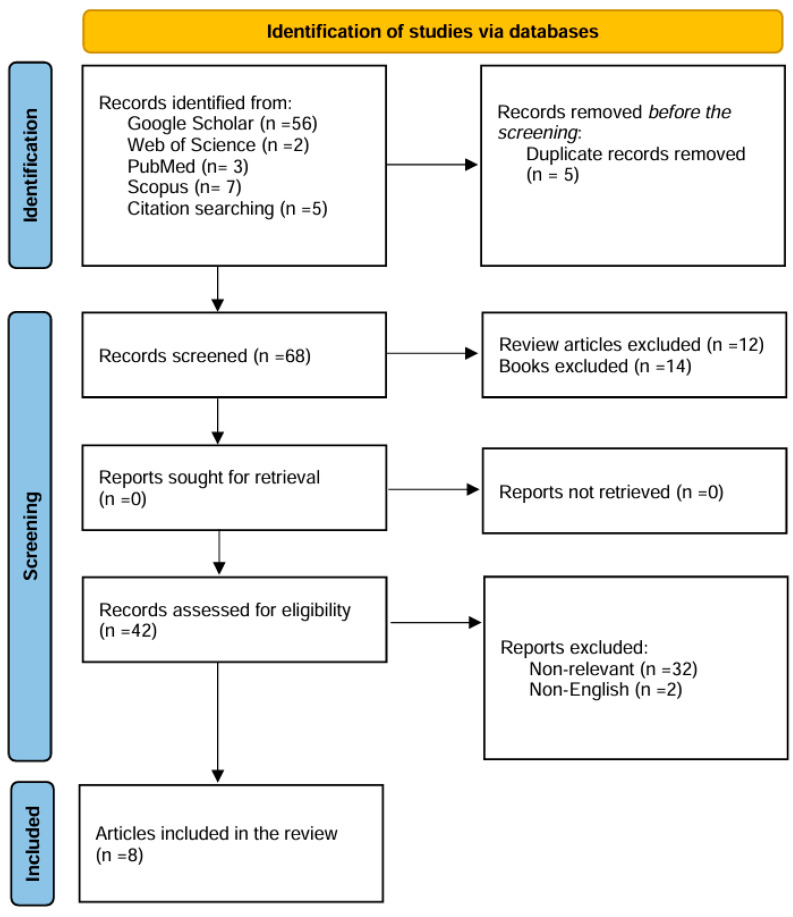
PRISMA flow diagram illustrating the systematic workflow for identifying studies on mitochondrial dysfunction in hypermobile Ehlers–Danlos Syndrome (hEDS) [28]. This figure outlines the systematic approach used to identify and evaluate relevant studies on the role of mitochondrial dysfunction in hEDS pathogenesis. The workflow begins with a comprehensive search across four major databases (PubMed, Web of Science, Google Scholar, and Scopus), yielding an initial pool of 68 articles. Following the removal of duplicates, reviews, and non-English publications, 42 primary research articles were screened for relevance and quality. After excluding non-relevant studies, a final selection of 8 original articles was included for in-depth analysis. Citation tracking was employed to ensure comprehensive coverage. This rigorous methodology highlights the limited but focused evidence base available for exploring mitochondrial dysfunction in hEDS.

The initial search yielded 73 articles. These were imported into Zotero version 7.0.11 (64-bit) for reference management. Duplicate entries were removed, reducing the dataset to 68 unique articles. A subsequent screening process excluded 12 review articles and 32 studies deemed non-relevant based on their titles and abstracts. Two articles not published in English were also excluded. This process resulted in a preliminary pool of four eligible studies. To ensure comprehensive coverage, citation tracking was performed on the selected studies, identifying an additional three relevant articles. This culminated in a final pool of eight original studies for detailed review and analysis. The study selection process is detailed in Figure 1, which follows the PRISMA flow diagram format to illustrate the identification, screening, eligibility, and inclusion of studies [28]. Articles were screened using Zotero for duplicates; titles and abstracts were reviewed for relevance, followed by full-text reviews to finalize eligible studies. The process for selecting sources of evidence involved several steps: A systematic search was conducted across four major databases: PubMed, Web of Science, Google Scholar, and Scopus. Duplicate entries were removed using Zotero reference management software. Titles and abstracts were screened for relevance based on predefined inclusion criteria, which focused on studies addressing mitochondrial dysfunction in hEDS or related pathways. Full-text articles were reviewed to confirm eligibility. Exclusion criteria included non-English publications, studies unrelated to hEDS or mitochondrial dysfunction, and reviews without original data. Citation tracking was performed on eligible studies to identify additional relevant articles.

### 2.2. Extraction and Analysis

Two independent reviewers extracted data from the selected studies using a standardized data extraction form. The extracted information included the following:

• Study design and methodology.
• Key findings related to mitochondrial dysfunction (e.g., OXPHOS defects, reactive oxygen species production).
• Genetic variations associated with mitochondrial or ECM pathways.
• Systemic manifestations linked to mitochondrial dysfunction in hEDS patients.
• Discrepancies between reviewers were resolved through discussion or consultation with a third reviewer.

### 2.3. Protein–Protein Interaction (PPI) Network Analysis

To explore the molecular interplay between mitochondrial dysfunction and non-mitochondrial pathways in hEDS, PPI network analyses using the STRING database (version 12) were performed. Identified genes from reviewed studies were mapped onto the network. We performed functional enrichment analysis to identify overrepresented biological processes and pathways, focusing on Gene Ontology (GO) terms related to mitochondrial function and cellular energetics. The STRING database integrates various sources of protein–protein interaction data, including experimental data, computational prediction methods, and public text collections, to create a comprehensive network of functional protein associations [29,30]. K-means clustering was applied to group genes into distinct functional clusters, providing insights into the interconnected nature of mitochondrial dysfunction in hEDS. The K-means algorithm partitions the genes into K clusters, where each gene belongs to the cluster with the nearest mean [29,30]. We iteratively adjusted the number of clusters (K) to optimize the separation of functional groups while maintaining biological relevance. This clustering approach allowed us to identify coherent groups of genes with similar functional properties or involvement in related biological processes. This network analysis approach allowed us to visualize and interpret the complex relationships between mitochondrial and non-mitochondrial pathways, offering a systems-level perspective on hEDS pathophysiology. The resulting clusters and enriched pathways provide a framework for understanding the multifaceted nature of mitochondrial involvement in hEDS and highlight potential areas for further investigation.

### 2.4. Comparative Analysis with Related Disorders

Insights from disorders with overlapping features, such as Fabry disease and fragile X premutation carriers, were integrated into the review. These conditions provided comparative data on shared mechanisms like RNA toxicity, ECM remodeling, and oxidative stress, offering additional context for understanding mitochondrial dysfunction in hEDS.

## 3. Results

This review synthesizes findings from recent studies to explore the intricate relationship between mitochondrial dysfunction and the pathogenesis of hEDS. Table 1 summarizes the key findings from the studies reviewed in this article.

### 3.1. Mitochondrial Dysfunction

Studies reveal that mitochondrial abnormalities disrupt OXPHOS and metabolic pathways, leading to bioenergetic deficits that may underlie key clinical manifestations such as chronic fatigue, muscle hypotonia, and systemic dysregulation [2,23]. Table 1 summarizes findings from studies exploring potential mitochondrial dysfunction in hEDS. These studies investigate various mechanisms, including altered energy metabolism (e.g., glycolysis and OXPHOS), lysosomal dysfunction, RNA toxicity, and mtDNA mutations. The findings highlight pathways such as fibroblast-to-myofibroblast transition (FMT), redox balance regulation, lysosomal dysfunction, ETC dysfunction, autophagy, and calcium dysregulation as potential contributors to hEDS pathogenesis. In this section, we detail the relationship between hEDS and potential mitochondrial dysfunction across these studied pathways.

#### 3.1.1. Mitochondrial Energy Metabolism

• Impaired OXPHOS: Proteomic analyses of hEDS fibroblasts have identified significant alterations in mitochondrial proteins critical for OXPHOS. The subunits of ATP synthase, including ATP5O, ATP5C1, and ATP5L, were found to be dysregulated, indicating compromised ATP production capacity [23]. Additionally, enzymes involved in the TCA cycle, such as aconitase 2 (ACO2), fumarate hydratase (FH), and malate dehydrogenase 2 (MDH2), exhibited altered expression levels in hEDS cells [23]. Studies have demonstrated that genetic variations in genes such as *MT-CYB*, *MT-ND1*, and *ACAD9*, which are involved in the mitochondrial OXPHOS system, are associated with hEDS [2,32,33]. These genes play critical roles in mitochondrial energy metabolism, and their dysregulation may contribute to the bioenergetic deficits observed in hEDS [2,32,33]. Specifically, mutations in *MT-CYB* and *MT-ND1* can impair ETC function, while variations in *ACAD9* disrupt mitochondrial dynamics and fatty acid beta-oxidation [37]. These disruptions may suggest a reduced ability of mitochondria to generate sufficient energy to meet cellular demands, particularly in tissues with high energy requirements like muscles and connective tissues.
• Metabolic Reprogramming Toward Aerobic Glycolysis: Evidence suggests that hEDS fibroblasts undergo metabolic reprogramming characterized by a shift from OXPHOS to aerobic glycolysis [23]. The upregulation of glycolytic enzymes—including glyceraldehyde-3-phosphate dehydrogenase (GAPDH), pyruvate kinase M1/2 (PKM), and lactate dehydrogenase B (LDHB)—has been observed [23]. This shift may represent an adaptive response to mitochondrial inefficiency but results in increased lactate production and reduced glucose oxidation. Such metabolic changes are consistent with the “Warburg effect”, commonly seen in cells under stress or with altered energy metabolism [23,38,39].
• Fatty Acid Oxidation and Beta-Oxidation Deficits: Enzymes involved in fatty acid beta-oxidation, such as acyl-CoA dehydrogenase very long chain (ACADVL) and enoyl-CoA hydratase 1 (ECHS1), were also dysregulated in hEDS fibroblasts [23]. Impaired beta-oxidation limits the ability of cells to utilize fatty acids as an alternative energy source, further exacerbating energy deficits [26]. This limitation may contribute to the exercise intolerance and post-exertional malaise frequently reported by hEDS patients [25,40].
• Mitochondria–ER Crosstalk and Calcium Homeostasis: Mitochondria actively communicate with the endoplasmic reticulum (ER) to regulate calcium signaling and energy production [41]. In hEDS fibroblasts, the upregulation of voltage-dependent anion channels (VDAC1 and VDAC2), which mediate the calcium flux between the ER and mitochondria, suggests altered calcium homeostasis [23]. Dysregulated calcium signaling can impair mitochondrial function, leading to further reductions in ATP synthesis and disruptions in redox balance [23,42].

#### 3.1.2. Elevated ROS and S100A4

Studies reported that increased ROS production in hEDS fibroblasts may be due to mitochondrial dysfunction [23]. This was accompanied by the upregulation of antioxidant enzymes, including superoxide dismutase 1 (SOD1), catalase (CAT), and glutathione peroxidase 1 (GPX1), indicating a compensatory response to oxidative damage [23,43,44,45]. The ROS-mediated activation of MMPs, such as MMP9 [46], was linked to ECM disorganization [23]. hEDS myofibroblasts showed high levels of S100A4, which is implicated in the disease states of different proinflammatory conditions [23]. S100A4 can cause mitochondrial disruption and oxidative stress in mast cells [47]. Silencing S100A4 can inhibit mitochondrial complex I activity and reduce cellular ATP levels [48].

#### 3.1.3. Comparative Insights from Mitochondrial-Related Diseases

The findings of potential mitochondrial dysfunction in hEDS can be further contextualized by examining parallels with Fabry disease (FD), a lysosomal storage disorder characterized by the accumulation of glycosphingolipids such as globotriaosyl ceramide (Gb3) and its deacylated form, lyso-Gb3 [31,49]. In FD, mitochondrial dysfunction is a significant downstream consequence of lyso-Gb3 accumulation, which disrupts cellular homeostasis through multiple pathways [49,50,51]. Studies have shown that lyso-Gb3 accumulation impairs mitochondrial energy metabolism by interfering with the autophagy-lysosomal pathway, including the dysregulation of the mechanistic target of rapamycin (mTOR) signaling [50,51]. The inhibition of mTOR signaling in FD leads to reduced mitochondrial efficiency and diminished ATP production [51,52]. Additionally, lyso-Gb3 toxicity has been linked to lysosomal and endothelial impairments, further exacerbating mitochondrial dysfunction [51]. These abnormalities contribute to systemic manifestations in FD, including chronic fatigue, musculoskeletal pain, and connective tissue fragility [23,31]. The toxic effects of lyso-Gb3 also extend to fibroblasts, where it disrupts collagen synthesis and ECM integrity [23,51]. This disruption mirrors the ECM abnormalities observed in hEDS, suggesting a shared potential role for mitochondrial dysfunction in connective tissue pathologies. The insights from FD underscore the broader implications of mitochondrial dysfunction as a unifying mechanism in multisystemic disorders [23]. In both hEDS and FD, a potentially impaired mitochondrial function may contribute to bioenergetic deficits, oxidative stress, and tissue fragility [23,31]. These parallels highlight the need for further research into potential mitochondrial-targeted therapies that could benefit patients with hEDS and other mitochondrial-related diseases.

A recent study on fragile X premutation carriers (fXPCs) revealed molecular mechanisms that overlap with those implicated in hEDS, particularly concerning mitochondrial dysfunction and its downstream effects [35]. An expansion of CGG characterizes fragile X premutation repeats in the FMR1 gene, leading to elevated levels of FMR1 mRNA and subsequent RNA toxicity [35,53]. This RNA toxicity has been shown to disrupt cellular homeostasis through several pathways, including calcium dysregulation, mitochondrial dysfunction, the enhanced production of ROS, and inflammation [35,53].

#### 3.1.4. Systemic Manifestations Linked to Mitochondrial Dysfunction

Mitochondrial dysfunction in hEDS contributes to a wide range of systemic manifestations due to the potential role of mitochondria in energy production, calcium homeostasis, and redox balance. These effects are particularly pronounced in tissues with high energy demands or those reliant on precise cellular signaling. Below, we expand on key systemic manifestations linked to mitochondrial dysfunction, incorporating recent findings and adding bone fractures as another significant manifestation.

• Chronic Fatigue: Reduced ATP production due to impaired OXPHOS might be a hallmark of mitochondrial dysfunction in hEDS [23,32]. Proteomic analyses of hEDS fibroblasts have revealed the dysregulation of ATP synthase subunits and enzymes involved in the TCA cycle, such as ACO2 and FH, leading to bioenergetic deficits [23]. This energy insufficiency manifests as chronic fatigue and post-exertional malaise, symptoms frequently reported by hEDS patients [25,26,40]. Similar findings are observed in fXPCs, where RNA toxicity disrupts mitochondrial function and reduces energy availability [35]. These shared mechanisms highlight the central role of mitochondrial dysfunction in fatigue-related symptoms.
• Neurological Symptoms: Mitochondrial dysfunction may also contribute to neurological manifestations such as migraine headaches, ataxia, and neuropathic pain in hEDS [54,55]. Dysregulated calcium signaling between ER and mitochondria impairs neuronal signaling and ATP synthesis [56]. Elevated ROS levels further exacerbate neuroinflammation and oxidative damage, contributing to cognitive impairments and pain sensitivity [57]. In fXPCs, RNA toxicity-induced calcium dysregulation and ROS overproduction have been linked to similar neurological symptoms, including brain fog and chronic pain [35]. These findings suggest that potential mitochondrial dysfunction may underlie the overlapping neurological features observed in both conditions.
• Gastrointestinal Dysfunction: The gastrointestinal system is particularly vulnerable to mitochondrial dysfunction due to the high energy demands of smooth muscle cells and enteric neurons [58]. Impaired OXPHOS has been associated with gastrointestinal motility [59]. These symptoms are frequently observed in hEDS patients and contribute significantly to their reduced quality of life.
• Bone Fractures: Bone fragility is an emerging systemic manifestation that may be linked to mitochondrial dysfunction in hEDS [2]. Mitochondria play a critical role in maintaining bone homeostasis by regulating osteoblast activity, osteoclast resorption, and calcium metabolism [60,61,62]. Disrupted mitochondrial dynamics and reduced ATP production impair osteoblast differentiation and mineralization while promoting oxidative stress that accelerates bone resorption [63]. Studies have shown that hereditary connective tissue disorders like hEDS are associated with an increased risk of fractures due to compromised ECM integrity and reduced bone density [5,64,65]. Additionally, mitochondrial dysfunction contributes to osteoporosis by increasing ROS levels which disrupt the balance between bone formation and resorption [60,61,66,67]. This mechanism may explain the heightened vulnerability to fractures observed in hEDS patients.
• Connective Tissue Fragility: Mitochondrial dysfunction may play a potential role in disrupting ECM homeostasis, contributing to the hallmark features of hEDS such as joint hypermobility, skin hyperextensibility, and tissue fragility. Mitochondria are essential for energy production required during collagen biosynthesis [16,20,68]. Impaired OXPHOS in hEDS fibroblasts leads to reduced ATP availability [23], limiting the energy necessary for collagen production. Studies have shown that mitochondrial dysfunction also affects the post-translational modification of collagen, such as hydroxylation, which is critical for stabilizing the triple-helical structure of collagen molecules [24]. This results in weakened connective tissue that is more prone to mechanical stress and injury [68].

#### 3.1.5. Sex-Specific Differences

The predominance of hEDS in females may be partially explained by sex-specific bioenergetic differences mediated by mitochondrial genetics and hormonal influences [6,7,8,22]. Estrogen has been shown to modulate mitochondrial biogenesis and function through ERα signaling pathways [21]. This hormonal regulation may influence ECM turnover and connective tissue resilience, which may contribute to the higher prevalence of hEDS in females.

### 3.2. Genetic Variations and Non-Mitochondrial Molecular Pathways

Unlike other subtypes of EDS with well-defined genetic causes, hEDS currently lacks a single causative gene [10]. Instead, recent studies suggest that hEDS arises from the interplay of multiple genetic variants and molecular pathways, which collectively contribute to its pathogenesis. The suggested genes involved in hEDS are summarized in Table 2, which provides a comprehensive overview of the genes and molecular pathways implicated in the pathogenesis of hEDS. It categorizes the genes into functional groups, such as primary candidate genes, ECM-related genes, signaling pathway genes, cell–matrix interaction genes, immune system-related genes, DNA damage response/stress genes, and emerging candidates. Each gene is accompanied by a brief description of its role or function in relation to hEDS (Table 2).

#### 3.2.1. Primary Candidate Genes

Several genes have been identified as potential contributors to hEDS. For example, the *KLK15* gene has been associated with a missense variant (p.Gly226Asp) found in approximately 2% of hEDS patients, potentially affecting connective tissue structure [69]. Additionally, *MIA3*, a gene involved in collagen transport, has emerged as a promising candidate in recent studies [70]. Genome-wide linkage analyses have also implicated *LZTS1* in a small percentage of hEDS patients [71], while heterozygous mutations in *TNXB* are known to disrupt collagen organization and ECM integrity [72]. Furthermore, variants in *COL3A1*, such as the p.Gly637Ser mutation, have been linked to collagen synthesis abnormalities in a single family with the autosomal dominant hEDS phenotype that was identified which led to reduced collagen secretion and the over-modification of collagen [5,73].

#### 3.2.2. Collagen and ECM-Related Genes

ECM plays a central role in maintaining connective tissue integrity, and several collagen-related genes have been implicated in hEDS. Variants in *COL5A1* and *COL12A1* disrupt type V and XII collagen, respectively, leading to ECM instability [2,74,75]. Other genes like *HSPG2* and *POSTN* are involved in ECM maintenance, while *MMPs* such as *MMP1, MMP2, MMP4, MMP8, MMP13*, and *MMP25* regulate ECM turnover and remodeling [74].

#### 3.2.3. Signaling Pathway Genes

Disruptions in key signaling pathways have also been associated with hEDS. Genes such as *TGFB3, SMAD2*, and *TGFB2* regulate TGF-β signaling, which is critical for tissue repair and remodeling [74]. The mechanosensing gene *PIEZO1* has been linked to joint mobility abnormalities [74], while novel mechanisms involving genes like *ULK4* and *KCNH1* suggest additional roles in tissue maintenance [74]. The gene *SORBS3* has also been implicated in regulating ECM stiffness [74].

#### 3.2.4. Cell–Matrix Interaction Genes

Genes involved in cell adhesion to ECM are crucial for maintaining tissue integrity. For instance, integrin-related genes such as *ITGA3, ITGB4, ITGA8,* and *ITGAV* mediate cell–ECM adhesion and signaling pathways [74]. Additionally, genes like *ROBO4* and *PCNT* contribute to vascular stability and cell–matrix interactions [74].

#### 3.2.5. Immune System Genes

Immune dysregulation is often observed in hEDS patients, with several immune-related genes being implicated. For example, variants in the antigen-processing genes *HLA-B* and *HLA-DRB1* have been linked to immune system involvement in hEDS [2]. Additionally, the tryptase-encoding gene *TPSAB1* is associated with mast cell activation disorders commonly seen in hEDS patients [2,74].

#### 3.2.6. Emerging Candidate Genes

Recent studies have identified additional genes that may contribute to the molecular basis of hEDS. These include transcriptional regulators like *ZNF717*, which may influence connective tissue health, and ion transport-related genes like *RHBG*, which could impact tissue homeostasis [2,78]. Other emerging candidates include structural protein-related genes such as ALDH18A1 and signaling regulators like DSE, which require further investigation [74,79].

### 3.3. Protein–Protein Interaction Network Highlights Interplay Between Mitochondrial and Non-Mitochondrial Pathways in hEDS

**Figure 2:**
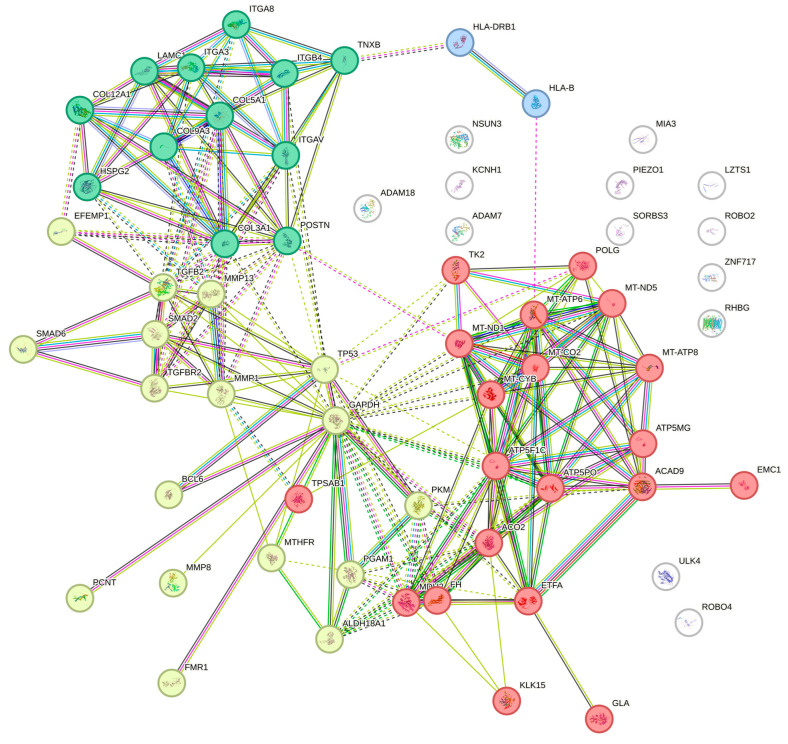
Protein–Protein interaction (PPI) network with functional clustering of mitochondrial and non-mitochondrial genes in hEDS. The PPI network depicted in this figure highlights the functional relationships among mitochondrial and non-mitochondrial genes implicated in hEDS. The network was analyzed using STRING [29,30], and the PPI enrichment *p*-value was calculated as <1.0 × 10^−16^, indicating that the observed interactions are significantly more frequent than expected by chance. This enrichment suggests that the proteins are biologically connected as a group, sharing common pathways or functions [29,30]. To further analyze the network, k-means clustering was applied, a machine learning algorithm that partitions data into distinct groups based on similarity. In this context, k-means clustering grouped the PPI network into four distinct clusters, each representing a unique set of biological processes: Cluster 1 (red nodes) includes mitochondrial genes such as MT-CYB, MT-ND5, and ATP5F1C, which are central to energy production through oxidative phosphorylation. These genes play a critical role in ATP synthesis and maintaining cellular bioenergetics. Cluster 2 (blue nodes) encompasses genes like COL3A1, COL5A1, and ITGB4, which are involved in extracellular matrix (ECM) remodeling, collagen biosynthesis, and integrin-mediated cell adhesion. These processes are essential for maintaining connective tissue integrity. Cluster 3 (yellow nodes) represents TGF-β signaling and elastic fiber formation. Genes such as TGFB2, SMAD2, and MMP13 dominate this cluster, highlighting their roles in TGF-β signaling, matrix metalloproteinase activity, and elastic fiber assembly. The dysregulation of these pathways contributes to tissue fragility and abnormal ECM turnover in hEDS. Cluster 4 (green nodes) includes immune-related genes like HLA-B and HLA-DRB1, which are involved in antigen presentation and immune system regulation. These interactions suggest a link between mitochondrial dysfunction and immune dysregulation commonly observed in hEDS patients. This clustering analysis provides a systems-level view of how mitochondrial dysfunction interacts with non-mitochondrial pathways to drive the pathophysiology of hEDS. It underscores the interconnected nature of energy metabolism, ECM remodeling, signaling pathways, and immune regulation in the disorder.

A PPI network analysis revealed significant interactions between mitochondrial genes (Table 1) and non-mitochondrial genes (Table 2), highlighting a complex interplay that may underlie the pathophysiology of hEDS (Figure 2) [29,30].

#### Functional Pathway Enrichment of PPI Network Clusters

To further elucidate the biological significance of the identified clusters in the PPI network [29,30], functional enrichment analysis was performed (Figure 2). This analysis aimed to identify overrepresented biological processes, molecular functions, and pathways associated with mitochondrial and non-mitochondrial genes implicated in hEDS. The results provide deeper insights into how mitochondrial dysfunction interacts with non-mitochondrial pathways to drive the systemic manifestations of hEDS. These interactions may suggest that mitochondrial dysfunction not only disrupts cellular energetics but also influences ECM remodeling, signaling pathways, and immune regulation through its crosstalk with non-mitochondrial pathways.

• Cluster 1 represents mitochondrial energy metabolism and oxidative stress. Functional enrichment revealed that genes in this cluster are predominantly involved in OXPHOS, ATP synthesis, and redox balance regulation. Key pathways include the following: The ETC, critical for ATP production. ROS detoxification processes, highlighting the role of antioxidant enzymes such as SOD2 and GPX1 in mitigating oxidative stress. These findings underscore the role of impaired mitochondrial bioenergetics in symptoms such as chronic fatigue and muscle hypotonia observed in hEDS (Figure 2; red nodes).
• Cluster 2 represents ECM remodeling and cell–matrix interactions. Genes in this cluster showed significant enrichment for ECM-related pathways, including collagen biosynthesis and post-translational modifications. ECM-receptor interaction pathways involving integrins like ITGB4 that mediate cell adhesion. The dysregulation of these processes may explain hallmark features of hEDS, such as joint hypermobility and tissue fragility (Figure 2; yellow nodes).
• Cluster 3 represents TGF-β signaling and ECM turnover. This cluster was enriched for pathways related to the following: TGF-β signaling, which regulates tissue repair and fibrosis. Matrix metalloproteinase (MMP) activity, particularly MMP13, which degrades ECM components like collagen. Elastic fiber formation is essential for maintaining connective tissue elasticity (Figure 2; green nodes). These findings suggest that mitochondrial dysfunction may amplify TGF-β signaling dysregulation, contributing to abnormal ECM turnover in hEDS.
• Cluster 4 represents immune regulation and antigen presentation. Enrichment analysis identified immune-related pathways, including antigen processing and presentation via major histocompatibility complex (MHC) proteins (HLA-B, HLA-DRB1) [2]. Peptide antigen binding, which may link mitochondrial dysfunction to immune dysregulation commonly observed in hEDS patients. This highlights a potential role for mitochondrial ROS in modulating immune responses and contributing to conditions like mast cell activation syndrome (MCAS) seen in hEDS (Figure 2; blue nodes).
• Emerging pathways of interest: Novel interactions identified in the PPI network suggest additional pathways requiring further investigation including ion transport regulation by genes like *RHBG*, which may influence tissue homeostasis. Transcriptional regulation by ZNF717 potentially affects gene expression patterns relevant to connective tissue health [2,77].

### 3.4. Therapeutic Implications: Targeting Mitochondrial Dysfunction in hEDS

Mitochondrial dysfunction, as highlighted in this review, may play a potential role in the pathophysiology of hEDS. This dysfunction could provide a basis for exploring therapeutic strategies aimed at alleviating the systemic manifestations of the disorder. While these approaches remain speculative and require further validation, they offer promising avenues for future research and clinical application.

#### 3.4.1. Enhancing Mitochondrial Bioenergetics

Impaired OXPHOS and reduced ATP production are hallmark features of mitochondrial dysfunction in mitochondrial dysfunction [80]. Strategies to support mitochondrial energy production might include the following:

• Coenzyme Q10 (CoQ10): As an electron carrier in the ETC, CoQ10 supplementation could improve mitochondrial efficiency and reduce fatigue, as observed in other mitochondrial disorders [81].
• Nicotinamide riboside (NR) or nicotinamide mononucleotide (NMN): These NAD+ precursors may enhance mitochondrial function by supporting cellular energy metabolism [82,83].
• Exercise-based interventions: Although exercise has been shown to promote mitochondrial biogenesis, its application in hEDS patients requires careful consideration due to the risk of post-exertional malaise [84,85].

#### 3.4.2. Reducing Oxidative Stress

Elevated ROS levels associated with mitochondrial dysfunction may contribute to ECM degradation and systemic inflammation in hEDS [86,87]. Antioxidant therapies could mitigate these effects:

• Mitochondria-targeted antioxidants: Compounds such as MitoQ or SkQ1 might specifically target mitochondrial ROS, reducing oxidative damage [88].
• Dietary antioxidants: Nutrients like vitamin C, vitamin E, and glutathione precursors could provide additional support for redox balance [89].

#### 3.4.3. Modulating ECM Remodeling

The interplay between mitochondrial dysfunction and ECM instability might be a key feature of hEDS. Therapies targeting ECM remodeling may help restore tissue integrity:

• Matrix metalloproteinase (MMP) inhibitors: These compounds are a family of zinc-dependent proteinases involved in the degradation of the ECM that could prevent excessive collagen degradation driven by ROS-mediated MMP activation [90,91,92].
• Tissue inhibitors of metalloproteinases (TIMPs): Enhancing TIMP activity might restore the balance between ECM synthesis and degradation [93].
• Collagen supplements or enhancers: These could support ECM repair by providing substrates for collagen biosynthesis [94].

## 4. Discussion

Unlike other EDS subtypes, hEDS lacks specific genetic markers, making it challenging to establish causative molecular pathways [2,5]. The absence of a single unifying genetic mutation suggests that hEDS may involve a multifactorial etiology, including both mitochondrial and non-mitochondrial mechanisms [2,78]. A recent survey revealed that individuals with clinically confirmed hEDS received an average of 10 years of alternative diagnoses during their diagnostic journey, with conditions such as anxiety, depression, and migraines being the most common [11]. This delay not only exacerbates patient distress but also delays appropriate management, potentially worsening outcomes. This underscores the challenge of distinguishing hEDS from other disorders with overlapping symptoms, such as fibromyalgia, functional neurological disorders, and multiple sclerosis [11]. Notably, many patients rejected these initial diagnoses upon receiving their hEDS diagnosis, reflecting a significant burden of misdiagnosis and prolonged diagnostic uncertainty [11]. The complexity of hEDS, involving multiple bodily systems and diverse manifestations such as chronic pain, dysautonomia, and gastrointestinal dysfunction, further complicates timely diagnosis [95]. Misdiagnosis can have profound consequences for vulnerable populations, particularly children [64,65,96,97]. Case studies have documented instances where symptoms of hEDS such as easy bruising and fractures due to connective tissue fragility were mistaken for signs of child abuse [64,65,96,97]. The uncertainty surrounding hEDS diagnosis can lead to significant psychosocial distress for patients [95]. A narrative review identified illness uncertainty (IU) as a key factor contributing to emotional distress in hEDS patients, driven by ambiguities in symptom attribution, diagnosis, and treatment options [95]. Studies suggest that healthcare providers may lack awareness of specific hEDS phenotypes or dismiss symptoms as psychosomatic [65,95,96,97]. This complexity underscores the need for more comprehensive genetic studies to identify candidate genes and their roles in hEDS pathogenesis.

This review underscores the hypothetical interplay between mitochondrial dysfunction and the systemic manifestations of hEDS. Mitochondrial dysfunction may act as a potential contributor to hEDS pathophysiology through several interconnected mechanisms [2]. OXPHOS and elevated reactive oxygen species levels could disrupt cellular energetics, leading to bioenergetic deficits that manifest as chronic fatigue and muscle hypotonia [98,99]. A meta-analysis of 17 patient cohorts with mitochondrial diseases suggests a potential link between OXPHOS defects and signs of hypermetabolism [98]. Clinically, these defects are associated with a broad range of multisystem disorders, including symptoms such as fatigue and exercise intolerance [98,100,101]. Consequently, many individuals with mitochondrial diseases tend to avoid physical activity and exercise, likely due to the exacerbation of these symptoms [98,102,103].

Furthermore, mitochondrial dysfunction may exacerbate ECM instability by influencing collagen biosynthesis and post-translational modifications [16,104,105], thereby possibly contributing to hallmark features of hEDS such as joint hypermobility, skin hyperextensibility, and tissue fragility. Mitochondria dysfunction influences ECM composition and vice versa [106]. Our review highlights potential crosstalk between mitochondrial abnormalities and non-mitochondrial pathways, including ECM remodeling, immune regulation, and signaling cascades (Figure 2). PPI network analyses suggest that mitochondrial dysfunction could amplify systemic manifestations by interacting with key molecular pathways, such as TGF-β signaling and matrix metalloproteinase activation (Figure 2). A recent study showed that the TGF-β receptor mediates communication between the ECM and mitochondria [16]. These interactions may further disrupt ECM homeostasis and tissue integrity [16]. Additionally, the systemic implications of mitochondrial dysfunction extend to neurological [107] and gastrointestinal symptoms [58] commonly observed in hEDS. Altered calcium homeostasis and increased ROS production may impair neuronal signaling and smooth muscle function [108], contributing to migraines [109], cognitive impairments [110,111], gastrointestinal dysmotility, and other multisystemic symptoms. The proposed relationship between mitochondrial dysfunction and these diverse clinical features provides a framework for future research aimed at elucidating the molecular underpinnings of hEDS.

While this review provides valuable insights into the potential role of mitochondrial dysfunction in the pathogenesis of hEDS, several limitations must be acknowledged. First, the evidence base is notably sparse, with only eight original studies directly addressing the relationship between mitochondria and hEDS. This limited dataset restricts the ability to draw robust conclusions and highlights the need for further research to substantiate these preliminary findings. A significant limitation in existing studies is the small sample sizes in genetic investigations, which reduce power and may fail to capture the full spectrum of genetic variability associated with hEDS. Many identified genetic variants lack functional validation, leaving their biological significance unclear. Without experimental confirmation, it remains speculative whether these variants directly contribute to mitochondrial dysfunction or broader hEDS pathophysiology. Additionally, while this review proposes mechanisms linking potential mitochondrial dysfunction to specific hEDS symptoms such as fatigue, connective tissue fragility, and gastrointestinal dysmotility, the exact molecular pathways remain poorly defined. The interplay between mitochondrial abnormalities and non-mitochondrial pathways, such as ECM remodeling and immune dysregulation, is suggested but not yet fully elucidated. This gap underscores the need for mechanistic studies using advanced tools like proteomics, bioinformatics, and functional assays. Another limitation is the lack of longitudinal data on disease progression in hEDS patients. Mitochondrial dysfunction may evolve over time or vary across different stages of hEDS, but current cross-sectional studies do not capture these dynamics. Longitudinal research could provide critical insights into how mitochondrial abnormalities contribute to disease onset, progression, and symptom variability. Finally, the heterogeneity of hEDS as a clinical entity presents challenges in interpreting findings. Future research should aim to standardize diagnostic frameworks and stratify patients based on molecular or clinical subtypes to improve study reproducibility and relevance. While these findings are based on emerging evidence and remain hypothetical, they emphasize the need for further investigation into the role of mitochondrial pathways in hEDS. To advance our understanding, future research should focus on the direct measurements of mitochondrial function and larger-scale genetic studies. Specifically, researchers should consider implementing techniques such as seahorse extracellular flux analysis to quantify the oxygen consumption rate (OCR) and extracellular acidification rate (ECAR) in patient-derived cells [112]. Additionally, assessing mitochondrial membrane potential using fluorescent dyes like JC-1 [113] or TMRE [114] in live cells from hEDS patients could provide valuable insights. Quantifying ATP production [115] in hEDS patient cells compared to controls using luminescence-based assays would further elucidate the role of mitochondrial pathways in hEDS pathogenesis. Analyzing mtDNA copy number and integrity through qPCR and long-range PCR techniques [116] in hEDS patient samples could reveal potential mitochondrial DNA alterations associated with the condition. Furthermore, investigating mitochondrial morphology and dynamics using high-resolution microscopy techniques in patient-derived cells would provide crucial information about the structural and functional changes in mitochondria [117] in hEDS.

Despite the limitations discussed, the novelty of this review lies in its pioneering exploration of mitochondrial dysfunction as a potential unifying mechanism in the pathophysiology of hEDS. This novel perspective opens a new era in hEDS research by integrating mitochondrial pathways into the broader understanding of this complex disorder. By proposing mitochondria as a potential player, this review provides a fresh framework for investigating hEDS, shifting the focus from solely ECM abnormalities to include cellular energetics and mitochondrial dynamics. The current review not only highlights the potential role of mitochondrial dysfunction in hEDS but also suggests that it may address several unresolved research gaps.

First, it offers an explanation for the wide spectrum of hEDS phenotypes, ranging from mild to severe. Variability in mitochondrial function across tissues and individuals could contribute to differences in symptom severity and progression [118]. Second, it provides a rationale for the lack of a single causative gene for hEDS. The interplay between multiple genes and their interaction with mitochondrial pathways or just mitochondrial genes may underlie the multifactorial nature of hEDS. Recent research suggests the potential role of mitochondrial DNA (mtDNA) mutations and mitochondrial dysfunctions in the development of connective tissue disorders [18,19,20]. A groundbreaking study by Schaefer et al. revealed that common mtDNA mutations, when present in an incompatible mtDNA background, can have far-reaching effects beyond the mitochondria themselves [20]. These mutations were found to alter the nuclear epigenome and influence the expression of numerous extracellular matrix genes [20]. This discovery provides a novel mechanism linking mitochondrial dysfunction to the widespread connective tissue abnormalities observed in disorders such as hEDS, opening new avenues for understanding the complex interplay between mitochondrial function and connective tissue homeostasis. This complexity suggests that future studies should prioritize examining mtDNA and its heteroplasmy through tissue-specific biopsies, rather than relying solely on genomic DNA from blood samples [119]. Such approaches may uncover tissue-specific mitochondrial dysfunctions that are masked in broader genomic analyses [120]. Additionally, our review proposes that the predominance of hEDS in females [6,7,8] could be partially explained by hormonal influences on mitochondrial function [21,22]. Estrogen has been shown to regulate mitochondrial biogenesis and activity, potentially amplifying sex-specific differences in bioenergetics and ECM turnover [21,22]. This hypothesis warrants further investigation into the role of sex hormones in modulating mitochondrial pathways and their contribution to hEDS pathophysiology. Importantly, the current review lays the groundwork for future research directions. It encourages studies aimed at validating the proposed links between mitochondria and hEDS through functional assays, advanced proteomics, and longitudinal analyses. Furthermore, it highlights the potential for clinical trials targeting mitochondrial function as a therapeutic strategy. Interventions such as mitochondrial antioxidants, coenzyme Q10 supplementation, or NAD+ precursors could be explored as potential treatments to alleviate systemic manifestations like fatigue, connective tissue fragility, and dysautonomia.

## 5. Conclusions

This review highlights mitochondrial dysfunction as a potentially unifying mechanism in the complex pathophysiology of hEDS. By integrating evidence from mitochondrial biology, ECM remodeling, and systemic manifestations, this work provides a novel framework for understanding the multifactorial etiology of hEDS. Impaired OXPHOS, elevated ROS, and calcium dysregulation emerge as central contributors to cellular energetics deficits and ECM instability, which may underlie hallmark features of hEDS such as joint hypermobility, skin hyperextensibility, and systemic symptoms like chronic fatigue and gastrointestinal dysfunction. Despite the limitations of the current evidence base such as small sample sizes, a lack of functional validation for identified genetic variants, and limited longitudinal data, this review opens new avenues for research. It addresses key gaps in hEDS studies by proposing that potential mitochondrial dysfunction could explain the wide phenotypic spectrum of hEDS, the absence of a single causative gene, and the predominance of female patients due to hormonal influences on mitochondrial function. These insights suggest that future investigations should prioritize tissue-specific mitochondrial analyses, including assessments of mtDNA heteroplasmy and functional studies in relevant tissues. The proposed relationship between mitochondria and hEDS not only advances our understanding of its molecular basis but also sets the stage for clinical trials that could transform patient care.

In conclusion, this work represents a significant step forward in unraveling the molecular complexity of hEDS. By bridging mitochondrial dysfunction, it lays the groundwork for a more comprehensive understanding of hEDS pathogenesis while offering a promising direction for future research and therapeutic development.

## Figures and Tables

**Table 1:** Summary of reviewed studies on potential mitochondrial dysfunction in hypermobile Ehlers–Danlos Syndrome (hEDS).

Reference	Sample Size (hEDS)	Potential Mitochondrial Dysfunction ^1^	Proteins/Genes ^1^	Measurements	Suggested Pathways
Shirvani et al. (2024) [2]	18 hEDS and 7 first-degree relatives as controls	Mitochondrial complex I assembly model OXPHOS system	*MT-CYB, MT-ND1, EMC1,* and *ACAD9*.	Whole-genome sequencing	Electron transport chain dysfunction
Chiarelli et al. (2021) [23]	Six hEDS dermal myofibroblasts were compared to 12 control fibroblasts	Altered glycolysis, citric acid cycle, respiratory electron transport and oxidative phosphorylation, increased oxidative stress, and impaired protein folding	GAPDH, PGAM1, PKM, LDHB, ATP5MG, ATP5O, ATP5F1C, FH, MDH2, ACO2, and ETFA	Proteome profiling/dermal myofibroblast pathways	Fibroblast-to-myofibroblast transition (FMT), integrin-linked signaling, and redox balance
Barbey et al. (2019) [31]	24 Fabry patients with the diagnostic criteria of hEDS	Lyso-Gb3 accumulation linked to impaired collagen synthesis, suggesting mitochondrial toxicity	*GLA* mutation and plasma lyso-Gb3	Genetic mutations and plasma evaluation	Lysosomal dysfunction pathways
Wilson and Tonk, (2024) [32,33]	834 hEDS of 1261 EDS patients	Mitochondrial depletion	*POLG/MT-DNA, MT-CO2, MT-TK*, and *MT-ND5, MT-RNA1/2, MT-tRNA, MT-ATP6/8, MT-CYB*	Clinical genomic analysis	Mitochondrial respiratory chain and mitochondrial ribosomal–transfer RNA
Wilson and Tonk, (2020) [34]	A case report of Mitochondrial Dysfunction and dysautonomia symptoms with history of EDS	Possibly complex V	*MT-ATP6, PLOD1, FLNA*, and *ATP2A*	Mt DNA and whole exome sequencing in blood and Mt DNA qPCR analysis in muscle biopsy	Mitochondrial respiratory chain and ATP synthesis
Tassanakijpanich et al. (2022) [35]	Five FMR1 fXPCs cases with characteristics of hEDS	Mitochondrial dysfunction and inflammation	*FMR1* gene	CGG repeat range (55–200 repeats) of the FMR1 gene	RNA toxicity also leads to Ca^2+^ dysregulation, mitochondrial dysfunction, enhanced production of reactive oxygen species, and inflammation
Jahanbani et al. (2024) [36]	A case report of a ME/CFS patient with hypermobility spectrum disorder and with a family history of hEDS	Mitochondrial respiration	TP53	Longitudinal cytokine profiling	Mitochondrial respiration, autophagy, and stress response

This table summarizes findings from studies exploring potential mitochondrial dysfunction in hypermobile Ehlers–Danlos Syndrome (hEDS). These studies investigate various mechanisms, including altered energy metabolism (e.g., glycolysis and oxidative phosphorylation), lysosomal dysfunction, RNA toxicity, and mtDNA mutations. The findings highlight pathways such as fibroblast-to-myofibroblast transition (FMT), redox balance regulation, lysosomal dysfunction, electron transport chain (ETC) dysfunction, autophagy, and calcium dysregulation as potential contributors to hEDS pathogenesis. ^1^ Abbreviations and key terms: GAPDH: Glyceraldehyde-3-phosphate dehydrogenase; a key enzyme in glycolysis. PGAM1: Phosphoglycerate mutase 1; involved in glycolysis. PKM: Pyruvate kinase M1/2; catalyzes the final step of glycolysis. LDHB: Lactate dehydrogenase B; converts pyruvate to lactate during anaerobic metabolism. ATP5MG, ATP5O, and ATP5F1C: Subunits of ATP synthase, essential for oxidative phosphorylation in mitochondria. FH: Fumarate hydratase; an enzyme in the tricarboxylic acid (TCA) cycle. MDH2: Malate dehydrogenase 2; catalyzes the conversion of malate to oxaloacetate in the TCA cycle. ACO2: Aconitase 2; involved in the TCA cycle. ETFA: Electron transfer flavoprotein subunit alpha; participates in mitochondrial fatty acid oxidation. lyso-Gb3: Globotriaosylsphingosine. *POLG*: Mitochondrial DNA polymerase gamma. *GLA*: Gene encoding α-galactosidase A; mutations of which cause Fabry disease and potentially are linked to mitochondrial dysfunction and impaired collagen synthesis. *FMR1*: Fragile X mental retardation 1 gene; associated with mitochondrial dysfunction and inflammation in fragile X premutation carriers (fXPCs). *MT-CYB, MT-ND1, MT-ND5, MT-RNA1/2, MT-tRNA*, and *MT-ATP6/8*: Mitochondrial genes involved in ETC function and mitochondrial ribosomal RNA synthesis. ME/CFS: Myalgic encephalomyelitis/chronic fatigue syndrome.

**Table 2:** Genes and molecular pathways associated with hypermobile Ehlers–Danlos Syndrome (hEDS).

Category	Gene	Function/Role
Primary Candidate	*KLK15*	Missense variant (p.Gly226Asp) found in ~2% of hEDS patients; affects connective tissue structure [69].
	*MIA3*	Implicated in collagen transport; identified as a promising candidate [70].
	*LZTS1*	Identified through genome-wide linkage analysis; linked to hEDS in some families [71].
	*TNXB*	Heterozygous mutations affecting collagen organization and ECM integrity [72].
	*COL3A1*	Includes variants like p.Gly637Ser, affecting collagen synthesis and organization [5,73].
Collagen and ECM	*COL5A1, COL12A1*	Variants affecting type V collagen and ECM structure [38,74,75].
	*HSPG2, POSTN*	Involved in ECM maintenance and structural stability [74].
	*MMP1, MMP8, MMP13*	Matrix metalloproteinases regulating ECM turnover and remodeling [74].
	*LAMC1, ROBO2*	Play roles in ECM organization and vascular integrity [74].
	*ADAM7, ADAM27*	Implicated in ECM remodeling processes [74].
Signaling Pathway	*TGFB3, SMAD2, SMAD6, TGFB2*	Key regulators of TGF-β signaling involved in tissue repair and remodeling [74].
	*PIEZO1*	Associated with mechanosensing and joint mobility [74].
	*ULK4, KCNH1*	Linked to novel mechanisms in tissue maintenance and cellular signaling.
	*SORBS3*	Regulates ECM stiffness and cellular mechanotransduction [74].
Cell–Matrix Interaction	*ITGA3, ITGB4, ITGA8, ITGAV*	Mediate cell adhesion to the ECM and integrin signaling pathways [76].
	*ROBO4, PCNT*	Associated with vascular stability and cell–matrix interactions [74].
Immune System	*HLA-B, HLA-DRB1*	Involved in antigen processing pathways; linked to immune dysregulation in hEDS.
	*TPSAB1*	Encodes alpha-tryptase; associated with mast cell activation disorders seen in hEDS patients [2,76].
DNA Damage/Stress	*BCL6, TP53*	Involved in DNA damage responses and cellular stress regulation mechanisms [36].
Other ECM Proteins	*COL9A3*	Affects collagen integrity; potential contributor to hEDS features.
	*ALDH18A1, EFEMP1*	Associated with structural stability of connective tissues [74].
Emerging Candidates	*ZNF717*	Emerging evidence suggests a role in transcriptional regulation linked to connective tissue health [77].
	*RHBG*	Potential involvement in cellular ion transport mechanisms relevant to tissue homeostasis [77].

## Data Availability

No new data were created.

## Supplementary Materials

Supplementary-PRISMA-ScR-Checklist. The supporting information can be downloaded at: https://www.mdpi.com/article/10.3390/cimb47020134/s1.
