# Supplementary material for: Mitochondrial Dysfunction and Its Potential Molecular Interplay in Hypermobile Ehlers–Danlos Syndrome: A Scoping Review Bridging Cellular Energetics and Genetic Pathways
Source: Curr Issues Mol Biol. 2025 Feb 19;47(2):134. doi: 10.3390/cimb47020134 (PMC11854588; doi:10.3390/cimb47020134)
Supplement: Supplementary file 1 [file cimb-47-00134-s001.zip › cimb-3442233-supplementary.pdf]

Preferred Reporting Items for Systematic reviews and Meta-Analyses extension for Scoping Reviews (PRISMA-ScR) Checklist

SECTION	ITEM	PRISMA-ScR CHECKLIST ITEM	REPORTED ON PAGE #
**TITLE**			
Title	1	Identify the report as a scoping review.	The title clearly identifies the report as a scoping review by its exploratory nature and broad focus on mitochondrial dysfunction in hEDS.
**ABSTRACT**			
Structured summary	2	Provide a structured summary that includes (as applicable): background, objectives, eligibility criteria, sources of evidence, charting methods, results, and conclusions that relate to the review questions and objectives.	The abstract provides a structured summary, including the background (mitochondrial dysfunction in hEDS), objectives (exploring potential links), methods (literature review and PPI analysis), results, and conclusions.
**INTRODUCTION**			
Rationale	3	Describe the rationale for the review in the context of what is already known. Explain why the review questions/objectives lend themselves to a scoping review approach.	The introduction explains the lack of definitive genetic markers for hEDS and highlights mitochondrial dysfunction as a novel area of exploration, providing strong rationale for the review.
Objectives	4	Provide an explicit statement of the questions and objectives being addressed with reference to their key elements (e.g., population or participants, concepts, and context) or other relevant key elements used to conceptualize the review questions and/or objectives.	The objective is to explore mitochondrial dysfunction as a unifying mechanism in hEDS pathophysiology and identify gaps for future research.
**METHODS**			
Protocol and registration	5	Indicate whether a review protocol exists; state if and where it can be accessed (e.g., a Web address); and if available, provide registration information, including the registration number.	No formal review protocol was developed or registered for this scoping review.
Eligibility criteria	6	Specify characteristics of the sources of evidence used as eligibility criteria (e.g., years considered, language, and publication status), and provide a rationale.	Inclusion criteria focus on studies addressing mitochondrial dysfunction in hEDS or related pathways; exclusion criteria include non-English articles and unrelated EDS subtypes.
Information sources*	7	Describe all information sources in the search (e.g., databases with dates of coverage and contact with	Databases searched include PubMed, Web of Science, Google Scholar, and Scopus without date restrictions but

SECTION	ITEM	PRISMA-ScR CHECKLIST ITEM	REPORTED ON PAGE #
		authors to identify additional sources), as well as the date the most recent search was executed.	limited to peer-reviewed English articles.
Search	8	Present the full electronic search strategy for at least 1 database, including any limits used, such that it could be repeated.	Articles were screened using Zotero for duplicates; titles and abstracts were reviewed for relevance, followed by full-text reviews to finalize eligible studies.
Selection of sources of evidence†	9	State the process for selecting sources of evidence (i.e., screening and eligibility) included in the scoping review.	The process for selecting sources of evidence involved several steps:<br>1. A systematic search was conducted across four major databases: PubMed, Web of Science, Google Scholar, and Scopus.<br>2. Duplicate entries were removed using Zotero reference management software.<br>3. Titles and abstracts were screened for relevance based on predefined inclusion criteria, which focused on studies addressing mitochondrial dysfunction in hEDS or related pathways.<br>4. Full-text articles were reviewed to confirm eligibility. Exclusion criteria included non-English publications, studies unrelated to hEDS or mitochondrial dysfunction, and reviews without original data.<br>5. Citation tracking was performed on eligible studies to identify additional relevant articles.<br>6. Seven original studies were included after this rigorous screening process.

SECTION	ITEM	PRISMA-ScR CHECKLIST ITEM	REPORTED ON PAGE #
Data charting process‡	10	Describe the methods of charting data from the included sources of evidence (e.g., calibrated forms or forms that have been tested by the team before their use, and whether data charting was done independently or in duplicate) and any processes for obtaining and confirming data from investigators.	Data extraction was conducted by two reviewers using a standardized form to collect information on study design, findings, genetic variations, and systemic manifestations linked to mitochondrial dysfunction in hEDS.
Data items	11	List and define all variables for which data were sought and any assumptions and simplifications made.	Variables included study design, mitochondrial dysfunction mechanisms (e.g., OXPHOS defects), genetic variants, systemic symptoms (e.g., fatigue), and pathways identified.
Critical appraisal of individual sources of evidence§	12	If done, provide a rationale for conducting a critical appraisal of included sources of evidence; describe the methods used and how this information was used in any data synthesis (if appropriate).	Critical appraisal of individual sources of evidence was not conducted in this scoping review. The primary objective was to map the breadth of existing literature on mitochondrial dysfunction in hEDS rather than evaluate the quality or risk of bias within individual studies. This approach aligns with the exploratory nature of scoping reviews, which aim to identify gaps in knowledge and provide an overview of available evidence without synthesizing findings quantitatively.
Synthesis of results	13	Describe the methods of handling and summarizing the data that were charted.	Evidence was synthesized qualitatively; PPI network analysis was conducted using STRING to explore interactions between mitochondrial and non-mitochondrial pathways relevant to hEDS pathophysiology.
**RESULTS**			
Selection of sources of evidence	14	Give numbers of sources of evidence screened, assessed for eligibility, and included in the review, with reasons for exclusions at each stage, ideally using a flow diagram.	Seven original studies were included; details such as sample size, mitochondrial genes/proteins studied, and methodologies (e.g., proteomics)

SECTION	ITEM	PRISMA-ScR CHECKLIST ITEM	REPORTED ON PAGE #
			are summarized in Table 1 of the article.
Characteristics of sources of evidence	15	For each source of evidence, present characteristics for which data were charted and provide the citations.	Results highlight impaired OXPHOS, ROS elevation, ECM remodeling, and genetic variants as key findings linking mitochondrial dysfunction to hEDS features like fatigue, tissue fragility, and systemic symptoms.
Critical appraisal within sources of evidence	16	If done, present data on critical appraisal of included sources of evidence (see item 12).	As noted in item 12, no critical appraisal was performed for this scoping review. The focus was on comprehensively identifying and mapping relevant studies rather than assessing their methodological rigor.
Results of individual sources of evidence	17	For each included source of evidence, present the relevant data that were charted that relate to the review questions and objectives.	Relevant data from each included study were charted systematically using a standardized data extraction form. Key variables extracted included:<br>• Study design and methodology.<br>• Identified mitochondrial dysfunction mechanisms (e.g., oxidative phosphorylation defects, ROS production).<br>• Genetic variations associated with mitochondrial or extracellular matrix (ECM) pathways.<br>• Systemic manifestations linked to mitochondrial dysfunction in hEDS patients (e.g., fatigue, tissue fragility).<br>• Suggested molecular pathways based on findings (e.g., TGF-β signaling, ECM remodeling).

SECTION	ITEM	PRISMA-ScR CHECKLIST ITEM	REPORTED ON PAGE #
			A summary table (Table 1 in the manuscript) presents these findings in detail.
Synthesis of results	18	Summarize and/or present the charting results as they relate to the review questions and objectives.	The charting results revealed several key insights:<br>1. Mitochondrial dysfunction is implicated in hEDS through mechanisms such as impaired oxidative phosphorylation, elevated ROS levels, calcium dysregulation, and ECM instability.<br>2. Genetic variants identified include mitochondrial genes (e.g., MT-CYB, MT-ND1) and non-mitochondrial genes involved in ECM remodeling (e.g., COL3A1, TNXB).<br>3. Systemic manifestations such as chronic fatigue, gastrointestinal dysfunction, and connective tissue fragility may be linked to these mitochondrial abnormalities.<br>4. Protein-protein interaction network analysis highlighted crosstalk between mitochondrial pathways and non-mitochondrial processes like immune regulation and TGF-β signaling.<br>These results align with the review's objective to explore potential links between mitochondria and hEDS pathophysiology while identifying gaps for future research.
**DISCUSSION**			

St. Michael's
Inspired Care.
Inspiring Science.

SECTION	ITEM	PRISMA-ScR CHECKLIST ITEM	REPORTED ON PAGE #
Summary of evidence	19	Summarize the main results (including an overview of concepts, themes, and types of evidence available), link to the review questions and objectives, and consider the relevance to key groups.	The discussion emphasizes how mitochondrial dysfunction could explain hEDS variability, lack of single causative genes, female predominance due to hormonal effects, and suggests future research directions such as tissue-specific analyses and clinical trials.
Limitations	20	Discuss the limitations of the scoping review process.	This scoping review has several limitations:<br>1. The evidence base is sparse, with only seven original studies directly addressing mitochondrial dysfunction in hEDS.<br>2. Small sample sizes in genetic studies limit generalizability and statistical power.<br>3. Many identified genetic variants lack functional validation, leaving their biological significance speculative.<br>4. The absence of longitudinal data restricts insights into how mitochondrial dysfunction evolves over time or contributes to disease progression.<br>5. The heterogeneity of hEDS as a clinical entity complicates efforts to establish consistent mechanisms or findings across studies.<br>6. No critical appraisal was performed due to the exploratory nature of this review.<br>These limitations highlight the need for more robust experimental studies, larger patient cohorts, and

SECTION	ITEM	PRISMA-ScR CHECKLIST ITEM	REPORTED ON PAGE #
			longitudinal analyses to validate proposed mechanisms.
Conclusions	21	Provide a general interpretation of the results with respect to the review questions and objectives, as well as potential implications and/or next steps.	This scoping review provides a novel framework for understanding hypermobile Ehlers-Danlos Syndrome (hEDS) by exploring mitochondrial dysfunction as a potential unifying mechanism in its pathophysiology. The findings suggest that impaired oxidative phosphorylation, elevated ROS levels, calcium dysregulation, and ECM instability may contribute to hallmark features such as joint hypermobility, skin hyperextensibility, chronic fatigue, and multisystemic symptoms.The results also address key research gaps:<br>1. Variability in mitochondrial function may explain the wide phenotypic spectrum of hEDS.<br>2. The lack of a single causative gene may reflect interactions between multiple genes influencing mitochondria or ECM pathways.<br>3. Hormonal influences on mitochondria could explain sex-specific differences in hEDS prevalence.<br>Future research should prioritize tissue-specific analyses of mitochondrial DNA heteroplasmy using biopsies from affected tissues rather than relying solely on blood samples. Additionally, longitudinal studies are needed to track disease progression and validate hypothesized mechanisms. Finally, clinical trials targeting mitochondrial functionsuch as antioxidant therapies or NAD+ precursors could offer promising therapeutic avenues for

SECTION	ITEM	PRISMA-ScR CHECKLIST ITEM	REPORTED ON PAGE #
			alleviating systemic manifestations like fatigue and connective tissue fragility.
**FUNDING**			
Funding	22	Describe sources of funding for the included sources of evidence, as well as sources of funding for the scoping review. Describe the role of the funders of the scoping review.	The article states that no external funding was received for this research.

JBI = Joanna Briggs Institute; PRISMA-ScR = Preferred Reporting Items for Systematic reviews and Meta-Analyses extension for Scoping Reviews.
* Where *sources of evidence* (see second footnote) are compiled from, such as bibliographic databases, social media platforms, and Web sites.
† A more inclusive/heterogeneous term used to account for the different types of evidence or data sources (e.g., quantitative and/or qualitative research, expert opinion, and policy documents) that may be eligible in a scoping review as opposed to only studies. This is not to be confused with *information sources* (see first footnote).
‡ The frameworks by Arksey and O'Malley (6) and Levac and colleagues (7) and the JBI guidance (4, 5) refer to the process of data extraction in a scoping review as data charting.
§ The process of systematically examining research evidence to assess its validity, results, and relevance before using it to inform a decision. This term is used for items 12 and 19 instead of "risk of bias" (which is more applicable to systematic reviews of interventions) to include and acknowledge the various sources of evidence that may be used in a scoping review (e.g., quantitative and/or qualitative research, expert opinion, and policy document).

From: Tricco AC, Lillie E, Zarin W, O'Brien KK, Colquhoun H, Levac D, et al. PRISMA Extension for Scoping Reviews (PRISMAScR): Checklist and Explanation. Ann Intern Med. 2018;169:467–473. doi: 10.7326/M18-0850.
